# Cardiometabolic health, nativity status and cognition among Latinos in the Health and Retirement Study

**DOI:** 10.1093/geroni/igaf122.4081

**Published:** 2025-12-31

**Authors:** Kenya Luna, Lauren Brown

**Affiliations:** University of Southern California, Los Angeles, California, United States; University of Southern California, Los Angeles, California, United States

## Abstract

Cardiometabolic disease increases dementia risk for older adults. Latinos face a higher burden of cardiometabolic risk factors than their white counterparts. It is unclear how nativity or location of birth impacts the relationship between cardiometabolic disease and cognition among Latinos. Importantly, these are potentially modifiable factors that can reduce the risk of developing dementia later in life. This study examines cardiometabolic biomarkers among foreign-born and U.S. born Latinos and determines whether these biomarkers are associated with increased risk of cognitive decline. Using the 2016 Health and Retirement Study with venous blood biomarkers (n = 1027), descriptive statistics show that U.S. born Latinos had higher history of cardiometabolic diseases [1.29(0.08)], lower fasting glucose [121.52(4.10)], and higher hemoglobin [13.90(0.13)] than foreign born Latinos. OLS models show history of cardiometabolic risk (𝜷=-0.62; p < 0.01) predict worse cognitive function while higher hemoglobin levels (𝜷= 0.30; p < 0.01) predicts better cognitive function among both US and foreign-born Latino populations. However, nativity stratified OLS models show that cardiometabolic history (𝜷= -0.85; p < 0.01) predict worse cognitive function while glucose (𝜷= 0.01; p < 0.05) and hemoglobin (𝜷= 0.46; p < 0.01) predict better cognitive function among foreign-born Latinos. Cardiometabolic history (𝜷= -0.26; p = 0.36), glucose (𝜷= -0.01; p = 0.14) and hemoglobin (𝜷= 0.13; p = 0.42) were not significant predictors of cognitive function among U.S. Born Latinos. Findings highlight early risk factors of cognitive decline for Latinos vary by nativity. These differences may guide culturally and contextually relevant treatments among older Latino populations.

